# The Physical Activity Type Most Related to Cognitive Function and Quality of Life

**DOI:** 10.1155/2020/8856284

**Published:** 2020-12-18

**Authors:** Saud F. Alsubaie, Abdulaziz A. Alkathiry, Walid Kamal Abdelbasset, Gopal Nambi

**Affiliations:** ^1^Department of Health and Rehabilitation Sciences, College of Applied Medical Sciences, Prince Sattam Bin Abdulaziz University, Al-Kharj 16278, Saudi Arabia; ^2^Department of Physical Therapy, College of Applied Medical Sciences, Majmaah University, Majmaah 11952, Saudi Arabia

## Abstract

**Background:**

Physical activity has been found to maintain and improve cognitive function and consequently improve health-related quality of life (HRQoL). The relationships between different types of physical activities, cognitive function, and HRQoL have not been studied sufficiently and compared in different age and gender groups. This study is aimed at examining the relationship between different types of physical activity (high-intensity, moderate-intensity, and walking exercise), cognitive function, and HRQoL. In addition, this study is aimed at examining these relationships in different age and gender groups.

**Methods:**

This cross-sectional study included 150 adults with a mean age of 50 ± 8.8 years. Participants completed the International Physical Activity Questionnaire (IPAQ) to assess the level of the physical activity types and the Short-Form Health Survey (SF-36) questionnaire to assess HRQoL. Cognitive function was measured using the Montreal Cognitive Assessment (MoCA) screening instrument. Spearman correlation analysis was used to explore the relationships between the different variables of the study.

**Results:**

There were significant positive relationships between all types of physical activities, cognitive ability, and HRQoL. The relationships between moderate-intensity physical activities and cognitive function (*r* = 0.38) and HRQoL (*r* = 0.33) were higher than the relationships with walking exercise and high-intensity physical activity. The middle-aged group had a significantly higher cognitive function compared to the senior adults (*p* < 0.001), while there was no significant difference between the age groups in HRQoL (*p* = 0.18).

**Conclusion:**

The cognitive function and HRQoL were more related to moderate-intensity physical activities compared to walking exercise or high-intensity physical activities. These relationships were more pronounced in the senior adult population compared to the middle-aged group.

## 1. Introduction

The number of older adults has been growing in the last few decades according to global statistics and is predicted to reach around two billion in 2050 [1]. This increase in the older population imposes a heavy burden on health care services. The risk of falling increases as people get older [2], which can be a life-threatening issue as it might result in death or serious injuries, such as a hip fracture [2]. In addition, the older adults with a history of falling may develop a fear of falling which may result in decreased confidence and lower levels of participation in daily life activities [3]. Specifically, in Saudi Arabia, the average life expectancy increased from 64.4 in 1980 to 74.4 in 2015, according to a recent report from the United Nations [4]. This increase in life expectancy is attributed to the improvement of health services provided to individuals in Saudi Arabia, especially the elderly. As a result, the population rate for those over the age of 60 was 3% in 2010 and is expected to rise to 9.5 in 2035 [5].

Aging has been linked to reduced cognitive functions and low HRQoL [6, 7]. A number of studies suggest that this decline in cognitive activity and HRQoL may be associated with age-related biological changes and lack of physical and cognitive activities [8]. Therefore, it is necessary to develop activities and strategies to improve cognitive function and slow down its decline. Several therapies have been suggested to improve cognitive function and limit its decline over time. Some studies have suggested customized approaches to treat risk factors associated with decreased cognitive function, such as diabetes and obesity, and found those approaches promising [9]. Some other studies have suggested the traditional Mediterranean diet and found promising results [10, 11]. Several studies including a World Health Organization (WHO) report indicated beneficial effects of physical activities on cognitive function [12–14] and HRQoL [8, 15]. It is sufficiently proven that exercises, especially aerobic exercises, lead to healthy aging, including the preservation of cognitive function [13, 14].

Although many studies have examined the relationship between physical activity, cognitive function, and HRQoL, these studies have not sufficiently studied which types of physical activity are most closely related to cognitive function and quality of life. Therefore, this study is aimed at examining the relationship between the time spent practicing different types of physical activity, cognitive function, and HRQoL to determine which types of physical activity are closely related to cognitive function and quality of life. Additionally, this study is aimed at examining these relationships in different age and gender groups.

## 2. Methods

### 2.1. Study Sample

One hundred-fifty adults aged between 40 and 86 years with a mean age of 50 ± 8.8 years were consecutively recruited. Participants were community-dwelling adults over the age of 40 from the Riyadh region. Participants were excluded if they were unable to move independently and required assistance from another person or device, if they had cardiovascular and neurological disease, or if they had a severe hearing, visual, speech, or cognitive impairments that prevented them from responding adequately to the examiner's questions. This study was approved by the Research Ethics Committee at Prince Sattam Bin Abdulaziz University (No. RHPT/018/004) in accordance with the guidelines of the Helsinki Declaration for medical research involving human subjects. Each participant provided their written consent before taking part in the study. The study took place from January to June 2018.

### 2.2. Sample Size

The required sample size was 120 subjects, based on an alpha level of 0.05, a power of 0.80, and an estimated effect size of 0.25 of the relationship between the variables of the study. The sample size was calculated using the G Power 3.1.7 software [16].

### 2.3. Study Design and Outcome Measures

This study was of a cross-sectional nature. Participants were asked to fill out two self-reported questionnaires to assess HRQoL and physical activity levels. In addition, a therapist administered a test to examine cognitive function.

HRQoL was assessed utilizing the Arabic version of the Short-Form Health Survey (SF-36) questionnaire including 36 items that examine eight domains of health status: general health, body pain, physical activity, physical function, mental health, social function, emotion status, and vitality. The total score of the questionnaire was 100 points, with a higher score indicating a better HRQoL [17].

Physical activity levels were evaluated utilizing the Arabic version of the International Physical Activity Questionnaire (IPAQ) [18]. The IPAQ is a self-reported questionnaire that determines the duration (time per day) and frequency (days per week) of the different types of physical activities in the last 7 days. The IPAQ categorizes physical activities into three categories, which are high-intensity physical activity, moderate-intensity physical activity, and walking exercise. The total number of hours and days for each physical activity category was calculated [19].

Cognitive function was examined utilizing the Arabic version of the Montreal Cognitive Assessment test (MoCA), for assessing mild cognitive disorder. MoCA examines various cognitive dimensions such as language, memory, orientation, calculations, conceptual thinking, executive functions, attention and concentration, and visual-constructional skills. The examiner administered the test, which took about 10 minutes to complete. The total score of the questionnaire was 30 points, with the participant being considered cognitively normal if the total score was 26 or higher [20].

### 2.4. Statistical Analysis

Demographic data and clinical characteristics included age, gender, body mass index, physical activity level, cognitive status, and HRQoL status and were summarized in descriptive statistics as means and standard deviations for quantitative variables or percentages for qualitative variables.

The distribution of the study variables was examined for normality using the Shapiro-Wilk test. It transpired that the variables of the study were continuous, but not normally distributed. Therefore, the Spearman correlation analysis was used to explore the relationship between the variables of the study. To assess the differences in study variables between the two age and gender groups, the Mann–Whitney *U* test was used. The analysis was performed using the Statistical Package for Social Sciences (SPSS software, Version 23).

## 3. Results

A total of 164 subjects were contacted for participation in the study. One-hundred fifty participants met the inclusion/exclusion criteria and agreed to participate in the study, while 14 subjects did not agree to participate in the study. Of the included participants, 59% were males (*n* = 89), and the age range of all participants was between 40 and 86 years old, and the average age was 50 ± 8.8. The middle-aged group included 111 participants (45.7 ± 4.2) while there were 39 participants (62.2 ± 7.1) in the senior adults' group.  Table 1 shows the characteristics of the participants.

The middle-aged group had a significantly higher cognitive ability (25.4 ± 3.6) than the senior adults' group (22.9 ± 4.3) (*p* = 0.039). The overall HRQoL was not significantly different between the two age groups (*p* = 0.183). However, the physical functioning domain of the HRQoL was higher in the middle-aged group compared to the senior adults (*p* = 0.035). While men did not differ significantly from women in terms of cognitive function, the overall HRQoL was higher for men than for women (*p* = 0.011). In addition, men reported higher scores than women in the following domains of HRQoL: physical functioning, emotional well-being, social functioning, and pain (Table 2).

Spearman correlation analysis showed a significantly negative relationship (*p* < 0.05) between age and walking exercise, moderate-intensity physical activities, cognitive functioning, and HRQoL, with correlation coefficients of -0.162, -0.201, -0.250, and -0.178, respectively. In addition, cognitive function and HRQoL were positively and significantly correlated with all different types of physical activities (Table 3). Among the different types of physical activities, moderate-intensity physical activity was the highest in the correlation coefficients with age, cognitive function, and HRQoL (Table 3).

A separate analysis was performed to investigate the relationship between study variables for each age and gender group (Table 4). Spearman correlation analysis revealed that cognitive function was significantly associated with all different types of physical activity in both age groups, except for walking exercise in the older adults' group. HRQoL was significantly correlated with moderate-intensity and high-intensity physical activities in both age groups. In general, older adults had greater correlation coefficients compared to the middle-aged group in relationships of cognitive function and HRQoL with the different types of physical activity. Cognitive function and HRQoL were significantly associated with all types of physical activity in both gender groups, except for walking exercise in the female group (Table 4).

## 4. Discussion

This study is aimed at assessing the relationships between the time spent practicing different types of physical activities, cognitive function, and HRQoL to determine which types of physical activities are most related to cognitive function and HRQoL. Additionally, this study is aimed at examining these relationships in different age and gender groups.

One of the main results of this study was that cognitive function declined with aging. The middle-aged group had higher cognitive function compared to the senior adults' group showing a decline in cognitive function as people age, which is consistent with results from other studies [21, 22]. Similar to our findings, Won et al. reported a significant correlation between age and cognitive functioning, showing a decline in cognitive functioning with aging [23]. Several studies have reported age-related changes in the brain that may affect cognitive function [24, 25], which may explain the decline in cognition levels in the senior participants in this study compared to the middle-aged participants.

The results of our study also indicated that the level of HRQoL was not different in middle-aged and older adults, which may not go in line with the results of other studies [22]. The sample of senior adults included in this study may not have had many age-related morbidities compared to senior adults in other studies, which may explain the absence of a difference between the middle-aged and the elderly in the quality of life. The absence of a difference in the quality of life between age groups indicated that the increase in age alone might not necessarily affect the quality of life. Several studies have indicated that the increase in morbidity may negatively influence the quality of life [22, 26]. There is also evidence linking the reduction of quality of life in older adults to several factors associated with increased age, including lack of physical activity, use of multiple medications, a higher number of chronic diseases, less involvement in social activities or relations, and inadequate social support [22, 26].

In addition, the results of our study showed a decline in physical activity levels as people got older, which was in line with reports from other studies. A number of other investigations, including one systematic review, have demonstrated that older people are less active than younger populations [27, 28]. In addition, a number of older adults especially those with a previous history of falling and fractures may develop a fear of falling, which in turn leads to less involvement in daily life activities [3].

The results of our study indicated a significant positive relationship between types of physical activity and both cognitive functioning and HRQoL, which is in line with the results of other studies [23, 29]. Interestingly, the relationships of cognitive function and HRQoL with types of physical activity were slightly higher in the moderate-intensity physical activities compared to other types of physical activities. Meanwhile, some relationships between walking activity and both cognitive ability and HRQoL did not reach the level of significance. A number of previous studies, including a systematic review, indicated a positive effect of moderate-intensity physical activities on both cognitive ability and quality of life compared to other physical activity intensities [30–33]. On the other hand, a number of studies and reviews showed that exercising at low-intensity levels did not affect cognitive ability differently compared to the cognitive ability of a sedentary group [33–35]. Similarly, Zhang et al. conducted a large study of the elderly population to examine the relationship between cognitive function and many variables, including daily life activities, which are considered generally as low-intensity physical activities. However, they did not find a relationship between total score of activities of daily living and cognitive function [36]. Many studies have not reported superiority of high-intensity physical activities to moderate-intensity physical activities at improving cognitive ability but, on the contrary, suggest including moderate-intensity physical activities to improve mental function [33–35]. A number of studies conducted on humans and animals have indicated the ability of moderate-intensity physical activities to modulate the central nervous system, specifically the areas responsible for cognition by increasing the plasticity and forming new synapses, which plays an important role in improving cognitive function [37–39].

In general, when looking at the relationships of cognitive function and HRQoL with types of physical activity for each age group separately, we found the correlation coefficients were slightly higher in the senior adults' group compared to the middle-aged group. The increase in age may have produced greater variability in the scores of cognitive function as well as the measure of the quality of life, in addition to the ceiling effect in the cognitive function and the quality of life data in the middle-aged data, which may explain the increase in the coefficients of the relationships in senior adults compared to the middle-aged group.

The results of the study, when examining the relationships between the study variables for each gender, showed relatively similar results. However, it is worth noting that the relationship between the duration of walking exercise, cognitive function, and HRQoL in females did not reach the level of significance, unlike moderate- and high-intensity physical activities. Previous studies have not adequately looked at the effect of gender on the relationships between types of physical activities and both cognitive function and quality of life, which confirms the importance of studying this aspect in future studies.

### 4.1. Strengths and Limitations

Compared to other studies that investigated the relationship between physical activities and cognitive function and HRQoL, this study examined the different levels on physical activity intensities in relation to cognitive function and HRQoL. Moreover, this study is considered an entry point for later studies that may lead to determining the optimal type and intensity of physical activity in improving cognitive function and quality of life. However, this study involved a relatively small number of participants compared to other studies, which may reduce the power of the analysis of relationships between study variables, especially when comparing age and gender groups. Furthermore, one of the limitations of this study is that although this study looks at the relationship between numbers of variables, it does not necessarily determine the causality between these variables due to the nature of this study, as it was conducted at one point in time. The sample of senior adults in this study may not have had many age-related morbidities compared to senior adults in other studies, which may reduce the possibility of generalizing the results of this study to some senior adult populations. In addition, the MoCA test used in this study to assess the level of cognitive function does not include all parameters of cognition such as reaction time and response accuracy that are usually included in cognitive studies. Therefore, it is important that future studies include more comprehensive tests to assess the different aspects of cognition. In addition, the occupational background information of the participants was not collected in this study, which may have an impact on the outcomes of the variables.

## 5. Conclusion

This study revealed that moderate-intensity physical activities were more related to both cognitive function and HRQoL compared to walking exercise and high-intensity physical activities, especially in the senior adult population. Rehabilitation programs that aim at improving cognitive function should focus on moderate-intensity physical activities, especially with an increase in age.

## Figures and Tables

**Table 1:** Characteristics of participants (*n* = 150).

Demographic data	All participants
Age (years)	50.0 ± 8.8
Gender (%)	
Male (*n* = 89)	59.3%
Female (*n* = 61)	40.7%
Age range (years)	40–86
Middle-aged (*n* = 111)	40–54
Senior adults (*n* = 39)	55–85
Weight (kg)	77.9 ± 15.9
Height (cm)	166.4 ± 8.6
BMI	28.1 ± 5.4
Physical activity types	
Walking (min/week)	114.6 ± 225.1
Moderate (min/week)	148.1 ± 280.2
Vigorous (min/week)	321.3 ± 446.3
Cognitive function	25.0 ± 3.8
HRQoL	
Overall score	69.6 ± 15.6
Physical functioning	73.5 ± 22.9
Physical health	66.2 ± 37.1
Emotional problems	74.3 ± 37.2
Energy/fatigue	60.4 ± 18.5
Emotional well-being	72.7 ± 18.2
Social functioning	78.2 ± 21.6
Pain	69.9 ± 23.9
General health	63.8 ± 18.4

Abbreviations: kg: kilograms; cm: centimeters; BMI: body mass index; HRQoL: health-related quality of life.

**Table 2:** Comparison of study variables based on age and gender.

Demographic data	Middle-aged (*n* = 111)	Senior adults (*n* = 39)	*p*	Male (*n* = 89)	Female (*n* = 61)	*p*
Age (years)	45.7 ± 4.2	62.2 ± 7.1	<0.001^∗∗^	50.6 ± 9.7	49.1 ± 7.4	0.636
Weight (kg)	79.5 ± 17.2	74.9 ± 9.9	0.182	82.0 ± 15.6	73.0 ± 14.4	<0.001^∗∗^
Height (cm)	167.1 ± 8.6	164.5 ± 8.5	0.128	171.1 ± 6.7	159.6 ± 6.4	<0.001^∗∗^
BMI	28.4 ± 5.7	27.3 ± 4.3	0.386	27.8 ± 5.1	28.7 ± 5.8	0.314
Physical activity types						
Walking (min/week)	355.7 ± 479.8	223.3 ± 318.0	0.119	293.9 ± 395.7	361.3 ± 512.0	0.724
Moderate-intensity (min/week)	153.0 ± 268.7	134.1 ± 313.9	0.105	173.1 ± 314.6	111.6 ± 218.1	0.183
High-intensity (min/week)	116.4 ± 217.0	109.5 ± 249.5	0.687	116.7 ± 221.0	111.6 ± 218.1	0.816
Cognitive function	25.4 ± 3.5	23.8 ± 4.2	0.039^∗^	25.5 ± 3.6	24.4 ± 4.0	0.093
HRQoL						
Overall score	70.7 ± 14.8	66.8 ± 17.5	0.183	72.1 ± 15.9	66.0 ± 14.4	0.011^∗^
Physical functioning	75.9 ± 21.8	66.7 ± 24.8	0.035^∗^	77.7 ± 22.2	67.4 ± 22.7	0.001^∗∗^
Physical health	66.9 ± 36.9	64.1 ± 37.9	0.677	69.4 ± 37.1	61.4 ± 36.9	0.146
Emotional problems	75.4 ± 36.9	71.1 ± 38.1	0.391	74.0 ± 37.4	74.7 ± 37.1	0.993
Energy/fatigue	61.1 ± 17.5	58.2 ± 21.3	0.279	61.1 ± 19.2	59.2 ± 17.6	0.558
Emotional well-being	71.4 ± 18.5	76.4 ± 17.0	0.159	75.1 ± 18.1	69.1 ± 17.9	0.036^∗^
Social functioning	77.9 ± 21.9	79.1 ± 20.7	0.826	80.5 ± 22.4	74.8 ± 20.0	0.029^∗^
Pain	70.5 ± 23.8	68.0 ± 24.7	0.543	74.2 ± 24.2	63.5 ± 22.2	0.004^∗∗^
General health	63.9 ± 18.0	63.3 ± 19.7	0.788	64.4 ± 17.9	62.8 ± 19.3	0.844

Notes: ^∗^*p* < 0.05; ^∗∗^*p* < 0.01. Abbreviations: kg: kilograms; cm: centimeters; BMI: body mass index; HRQoL: health-related quality of life.

**Table 3:** Spearman correlation coefficients of relationships among study variables.

Study variables	Age	Cognitive function	Physical activity (walking)	Physical activity (moderate-intensity)	Physical activity (high-intensity)
Age	—	-0.250^∗∗^	-0.162^∗^	-0.201^∗^	-0.097
Cognitive function	-0.250^∗∗^	—	0.264^∗∗^	0.381^∗∗^	0.267^∗∗^
HRQoL	-0.178^∗^	0.345^∗∗^	0.193^∗^	0.326^∗∗^	0.244^∗∗^

Notes: ^∗∗^correlation is significant at the 0.01 level (2-tailed). ^∗^Correlation is significant at the 0.05 level (2-tailed). Abbreviations: HRQoL: health-related quality of life.

**Table 4:** Spearman correlation coefficients of relationships among study variables based on age and gender.

	Middle-aged (*n* = 111)	Senior adults (*n* = 39)	Male (*n* = 89)	Female (*n* = 61)
Cognitive function and physical activity (walking)	0.215^∗^	0.296	0.359^∗∗^	0.162
Cognitive function and physical activity (moderate-intensity)	0.343^∗∗^	0.394^∗^	0.366^∗∗^	0.380^∗∗^
Cognitive function and physical activity (high-intensity)	0.238^∗^	0.324^∗^	0.219^∗^	0.325^∗^
HRQoL and physical activity (walking)	0.162	0.242	0.278^∗∗^	0.116
HRQoL and physical activity (moderate-intensity)	0.264^∗∗^	0.403^∗^	0.337^∗∗^	0.269^∗^
HRQoL and physical activity (high-intensity)	0.198^∗^	0.359^∗^	0.247^∗^	0.268^∗^

Notes: ^∗∗^correlation is significant at the 0.01 level (2-tailed). ^∗^Correlation is significant at the 0.05 level (2-tailed). Abbreviations: *n*: sample size; HRQoL: health-related quality of life.

## Data Availability

The data is available upon reasonable request.
